# Supplementary material for: Parallel Losses of Blue Opsin Correlate with Compensatory Neofunctionalization of UV-Opsin Gene Duplicates in Aphids and Planthoppers
Source: Insects. 2023 Sep 20;14(9):774. doi: 10.3390/insects14090774 (PMC10531960; doi:10.3390/insects14090774)
Supplement: Supplementary file 1 [file insects-14-00774-s001.zip › Supplementary data file 4.pdf]

Supplementary Data File S4: Annotated alignment of aphid and planthopper UV-opsin protein sequences. See Figure 1 for species aphid and planthopper species abbreviations. Outgroup sequences: *Chrysochroa rajah* (Craj) UV1-anc (WCQ76393) and UV2-der (WCQ76394), *Frankliniella occidentalis* (Focc) (XP_026279762).

```
                                                                11                      11111
                 12                                      345678901                      23456
Btau_rhodopsin 1 MN--------------------------------------GTEGPNFYV----------------------PFSNK-------  16
Craj_UV2-anc   1 MHLTN----------------Y--S-VKFP-------------------------EAR--VM---D---SGHQLIAWNVPAAD  31
Adif_UV2-anc   1 MVWSVSLSAVANPPTMMRLINW--T-DHTIL--A---------------------EPR--IM---E---SRPGSMEWNISPEN  49
Craj_UV1-der   1 MKEMM--------------FNG--TFLDGPL--P---------------------QAR--VL---S---EERRLLGWNVDNSD  36
Adif_UV1-der   1 ML-----------------LNW--TAIDRPL--P---------------------QLG--GD--SG---HEVRLLGWNIPPSE  34
Focc_UV        1 MEYPESAD-C---------SNG--T-AWLPV--A---------------------EARIGSGVSGP---SEQRLLGWNVPPDE  44
Dcit_UV        1 MESDFY-------------LND--T-SIGPVALARVAAQQSFSVNEKLFVPIFSPRVAAQQSF-SV---NGKRMLGWNVPPED  63
Pcel_UV        1 -----------------------------------------------------------------------------------   0
Apis_UV-anc    1 MSPTLIMD-----------FNR--T-VSRPL--A---------------------QLG--LME-NE--VGETHLLGWNLQAED  41
Rmai_UV-anc    1 MSPTLIMD-----------FNR--T-VSRPL--P---------------------QLG--LME-NE--VGETHLLGWNLPAED  41
Mper_UV-anc    1 MSPTMIMD-----------FNR--T-VSRPL--Q---------------------QLG--LME-NE--VGETHLLGWNLPAED  41
Sfla_UV-anc    1 MFPALIMD-----------FNR--T-VSRPL--P---------------------QMG--SAD-DA--GGETHLLGWNLGAED  41
Cced_UV-anc    1 MMM---MD-----------FNR--T-VSKPL--P---------------------ELG--LLE-NEPGETGRQLLGWNLAAED  40
Apis_UV-der    1 MD-----------------FNR--S-VSRPL--S---------------------QLGSSFME-NE---EELQLMGWNLTPED  36
Rmai_UV-der    1 MD-----------------FNR--S-VSRPL--S---------------------QLGSSFME-NE---DELQLMGWNLTPED  36
Mperi_UV-der   1 MD-----------------FNR--S-VSRPL--S---------------------QLGSSFME-NE---DELQLMGWNLTPED  36
Sfla_UV-der    1 MD-----------------FNR--S-ISRTL--S---------------------QLG--FME-EE---PELHLMGWNLTPED  34
Cced_UV-der    1 MD-----------------FNG--S-VLRPL--P---------------------QLG--PMD-ND---EEAHLMGWNLTPED  34
Ncin_UV        1 MEFFNGAS-----------YNTSS-----PVAMA---------------------RMG--LG---A---GAPRMLGWNVPTED  38
Evit_UV        1 MEVFDGAN-----------YNASAA-LFQPQALP---------------------RLG--M----P---NGVQMLGWNVPTEE  41
Monu_UV        1 MEYLSGAD-----------YNASAA-LFQPQALP---------------------RLG--M----P---NGVQMLGWNVPTEE  41
Nlug_UV-anc    1 MEAFGGANNC---------PNG--T-VWGPQ--A---------------------S----F----R---TAQRSLIWNVPTDE  37
Lstr_UV-anc    1 MDVFGGAKNC---------PNE--T-VWGPQ--A---------------------S----F----R---TAQRSLIWNVPTEE  37
Sfur_UV-anc    1 MDVFGGAKNC---------PNE--T-VWGPQ--A---------------------S----F----R---TAQRSLIWNVPTEE  37
Nlug_UV-der    1 MEVFRGASDC---------PNE--S-VWGPQ--V---------------------S----F----R---TAERTLIWNVPLEE  37
Lstr_UV-der    1 MDVFGGAKNC---------PNE--T-VWGPQ--V---------------------S----F----R---TAERTLIWNVPLEE  37
Sfur_UV-der    1 MDVFSGAKNC---------PNE--T-VWGPQ--A---------------------S----F----R---TAERTLIWNVPLEE  37
```

```
                     111222222222233333333----3333334444444444455555555555666666666667777777777788888888888899999999
                     789012345678901234----567890123456789012345678901234567890123456789012345678901234567890123456
Btau_rhodopsin 17 TGVVRSPFEAPQYYLAEP----WQFSMLAAYMFLLIMLGFPINFLTLYVTVQHKKLRTPLNYILLNLAVADLFMVFGGFTTTLY   96
Craj_UV2-anc   32 --L----EHIPEHWLKYPAPEASLHLLLGLLYIGLFLMSILGNGLVLWIFSSAKCLRTPSNMFIVNLAFCDFVMML-KTPIFIY  108
Adif_UV2-anc   50 --I----PYVPEHWLKFPPPEMSLNLLLGLLYIVFFFMSIIGNGLVIWIFSTAKSLRTPSNMFVVNLAFLDFVMML-KTPIFIF  126
Craj_UV1-der   37 --L----VHIPEHWLKYPEPEASIHFLLGLLYILYFLMSTIGNGLVLWIFSSSKSLRTPSNLFVVNLAFCDLIMLL-KAPLFVY  113
Adif_UV1-der   35 --L----VHIPEHWLKYPEPEASLHFLLILLYLICFLLSTVGNGMVIWIFSTAKSLRTPSNMFVVNLAVLDFIMLS KSPLFIY  111
Focc_UV        45 --L----PHIPTHWLVYPEPDPLINYGLGVLYCFFFFFAVMGNGIVIYIFLTSKNLKTPSNIFVVNLAICDFVMMA-KTPIFIY  121
Dcit_UV        64 --R----FRIPEHWFQYEEIDPMYNYVLGCLYIVFTIFSICGNGMVIWVFIAAKSLRTPSNIFVLNLALMDFMMMV-KTPIFIY  140
Pcel_UV        64 ----------------------MNNYILGSLYCVFCAVSLIGNGLVIWIFLTAKALRTPSNLFVVNLAIMDFVMML-KTPIFIY  161
Apis_UV-anc    42 --L----IHIPEHWLKYQEPSSLQHYYLAFMYTIFMFVALFGNGLVIWVFCVAKPLRTPSNIFVINLALCDFVMMA-KAPIFIL  118
Rmai_UV-anc    42 --L----IHIPEHWLKYQEPSALQHYYLAFMYTLFTFIALFGNGLVIWIFCIAKPLRTPSNIFVINLALCDFVMMA-KAPIFIY  118
Mper_UV-anc    42 --L----IHIPEHWLKYQEPSSLQHYYLAFMYTIFTFVALFGNGLVIWIFCIAKPLRTPSNIFVINLALCDFVMMA-KSPIFIY  118
Sfla_UV-anc    42 --L----IHIPEHWLKYEEPSALQHYYLAFMYTIFTIIALVGNGLVIWIFCISKSLRTPSNIFVINLALCDFLMMA-KAPIFIY  118
Cced_UV-anc    41 --L----VHIPDHWLNYQEPSSLSHYYLAFMYSIFTIFALVGNGLVIWIFCVAKPLRTPSNIFVINLALCDFCMMA-KAPIFIY  117
Apis_UV-der    37 --L----THIPEHWLSYPEVRSLYHYILAFSYTILFCLGVIGNGLVLWIFCVSKPLRTPSNLFVLNLALCDFSMVL-VLPILIY  113
Rmai_UV-der    37 --L----THIPEHWLSYPEVRSLYHYILAFAYTILFSLGVIGNGLVLWIFCVSKPLRTPSNLFVLNLALCDFSMVL-VLPILIY  113
Mper_UV-der    37 --L----THIPEHWLSYPEVRSLYHYILAFAYTILFCLGVIGNGLVLWIFCVSKPLRTPSNLFVLNLAMCDFSMVL-VLPILIY  113
Sfla_UV-der    35 --L----IHIPEHWLSYPEVRSIYHYILAFMYSILFSLGVIGNGLVLWIFCVSKPLRTPSNLFVLNLALCDFSMVL-VLPILIY  111
Cced_UV-der    35 --L----VHVPEHWLSYPEVRSMYHYIMAFSYSILFVIGIIGNGLVIWIFSLSKPLRTPSNLFVLNLALCDFSMVL-VLPILIY  111
Ncin_UV        39 --L----VHIPQHWLKYQAPEAMQHYMLGFIYIFFMIASLIGNGLVIWIFCGARNLRTPSNMFVINLAICDFMMML-KTPIFIY  115
Evit_UV        42 --L----VHIPQHWLKYPAPEPIQHYVLGFLYIFFMFAALFGNGLVIWIFCGAKNLRTPSNMFVINLALCDFLMMS-KTPIFIY  118
Monu_UV        42 --L----VHIPQHWLKYPAPEPIQHYVLGFLYIFFMFAALFGNGLVIWIFCGAKNLRTPSNMFVINLALCDFLMMS-KTPIFIY  118
Nlug_UV-anc    38 --L----ANIPEHWFNYPEPDPMYNYVLGLLYVFFMFFSLIGNGLVIWIFCSAKSLRTPSNMFVVNLALCDFMMMV-KTPIFIY  114
Lstr_UV-anc    38 --L----PHIPEHWFNYPEPDPMYNYVLGLLYVFFMFFSLVGNGLVIWIFCSAKSLRTPSNMFVVNLALCDFLMML-KTPIFIY  114
Sfur_UV-anc    38 --L----PHIPEHWFNYPEPDPMYNYVLGLLYVFFMFFSLVGNGLVIWIFCSAKSLRTPSNMFVVNLALCDFMMML-KTPIFIY  114
Nlug_UV-der    38 --L----EHVPEHWFNHPAPEPIYNYVLGLVYMFYMFFSFIGNGLVIWIFSSAKTLRTPSNMFVVNLAICDLLMMV-KIPIFIY  114
Lstr_UV-der    38 --L----EHVPEHWFNHPAPEPIYNYVLGIVYMFYMFFSFVGNGLVIWIFSSAKSLRTPSNMFVVNLAICDLLMMI-KIPIFIY  114
Sfur_UV-der    38 --L----EHVPEHWFNHPAPEPIYNYILGLVYMFYMFFSFVGNGLVIWIFSSAKSLRTPSNMFVVNLAICDLLMMV-KIPIFIY  114
```

```
                           1111111111111111111111111111111111111111111111111111111111111111111111111111-1111111
                        99990000000000111111111122222222223333333333444444444455555555556666666666777-7777777
                        78901234567890123456789012345678901234567890123456789012345678901234567890123456789012-3456789
Btau_rhodopsin   97 TSLHGYFVFGPTGCNLEGFFATLGGEIALWSLVVLAIERYVVVCKPMSNFRFGENHAIMGVAFTWVMALACAAPPL-VGWSRYI  179
Craj_UV2-anc    109 NSFNRGFAAGFVSCQVFGVMGTLSGIGAGMSNACIAYDRYHTITNPLEG-KLTRTKALVMILFVWTYTTPWAILPLFEIWGKFV  191
Adif_UV2-anc    127 NSFKQGFYLSMVWCYVFVIAGTYSGIGASMTNVCIAYDRYHTITNPMEG-RLTRTKALLMILFIYVYVTPFAILPYLKIWGNFI  209
Craj_UV1-der    114 NAFNRGYATGHIGCQIFAMVGSLSGIGAGTTNACIAYDRYTTITNPLEG-RLSKTKAVILIIFVWMYTMPWVVLPLLEIWSRFV  196
Adif_UV1-der    112 NAFHRGYAAGHLGCQIFGTIGTLSGIGAGMTDVCIAYDRYTTITNPLEG-RMSKIKAIIMIIFVWLYTLPWIILPFTEVWNRFV  194
Focc_UV         122 NSFNLGPASGPTACLVFATMGSFSGIGAGATNAVIAYDRYMTIAKPFGS-KMTRGKAIMIIFFLWMWVTPWVVFPATEIWGRFV  204
Dcit_UV         141 NSFNQGYALGHQGCQIFGLMGSISGIGQSATNVAIAYDRYRVIAKPMDG-RMSYSKALMILILIYCYVIPWAMFPYLEKWSRFV  223
Pcel_UV         162 NSFHQGYELGHQGCQWFGLMGAITGIGQSATNVAIAYDRYRVIAKPMDG-RMSFSKAFIILIMIYAYVLPWALFPYFEKWSRFV  144
Apis_UV-anc     119 GSINRGY-QGHFLCQLFGTAGAFSGIGASATNAAIAYDRFSTIAKPFDG-RMTYGRAFFLIICIWTYTLPWGLLPLTEKWNRYV  200
Rmai_UV-anc     119 SSINRGY-QGHFLCQLFGVAGAFSGLGASATNAAIAYDRFSTIAKPFDG-RMTYGRALFLIVCIWTYTLPWGILPLTEKWNRFV  200
Mper_UV-anc     119 SSINRGY-QGHFLCQLFGVAGAFSGLGASATNAAIAYDRFSTIAKPFDG-RMTYGRAFFLIICIWTYTLPWGLLPLTEKWNRYV  200
Sfla_UV-anc     119 GSVQRTY-QGHFLCQAFALAGTISGLGASITNAAIAYDRYSTIAKPFEG-RMTYGRAFFIMILIWAYVFPWCFLPATEKWNRYV  200
Cced_UV-anc     118 NSIQRGY-QGHLNCQIFAMAGSISGIGASATNAAIAYDRFSTIAKPFDG-RMTFGKAFILIICIWTYVLPWCFLPLTEKWNRFV  199
Apis_UV-der     114 DSIDHKY-PGHLQCQIFALCGSISGIGAGATNAAIAYDRYSTIAKPFEG-RMTYGKALILIICIWIYVLPWCLLPLTEKWNRFV  195
Rmai_UV-der     114 DSINHKY-PGHLQCQIFALCGSISGIGAGATNAAIAYDRYSTIAKPFEG-RMTYGKALILIICIWIYVLPWCILPLTEKWNRFV  195
Mper_UV-der     114 DSIDHKY-PGHLQCQIFALCGSISGIGAGATNAAIAYDRYSTIAKPFEG-RMTYGKALILIICIWIYVLPWCLLPLTEKWNRYV  195
Sfla_UV-der     112 DSINHKY-PGHLQCQIFALCGSISGIGAGATNAAIAYDRYSTIAKPFEG-RMTYGKALILIICIWMYVFPWCFLPLTEKWNRYV  193
Cced_UV-der     112 DSIDHKY-PGHLQCQIFALCGSISGIGAGATNAAIAYDRYSTIAKPFEG-RMTYGKALILIVCIWIWVFPWCYLPLSEKWNRYV  193
Ncin_UV         116 NSFNLGFALGQTGCQVFSIMGSLSGIGAAATNAAIAYDRYSTIARPLDG-KMSRGKAFLFILCIWAYVSPWVFLPATQTWGRYA  198
Evit_UV         119 NSFKLGFALGQFWCQVFAIAGSLSGIGAAATNAVIAYDRYSTIARPLDG-KMSKGKAFLFLLCIWIYTTPWVFLPATQTWGRYA  201
Monu_UV         119 NSFKLGFALGQFWCQVFAIAGSLSGIGAAATNAVIAYDRYSTIARPLDG-KMSKGKAFLFLLCIWIYTTPWVFLPATQTWGRYA  201
Nlug_UV-anc     115 NSFNLGFATGHLGCQIFATIGSFSGIGASATNAAIAYDRYNVIAKPLDG-RMSKGKAFLIILLIWAYVTPWSLMPLYGVWSRFV  197
Lstr_UV-anc     115 NSFNLGFASGHLGCQIFGTIGSFSGIGASATNAAIAYDRYNVIAKPFDG-KMSKGKAFLIILLIWAYVTPWSLMPLYGYWSRFV  197
Sfur_UV-anc     115 NSFNLGFASGHLGCQIFGTIGSFSGIGASATNAAIAYDRYNVIAKPFDG-KMSKGKAFLIILLIWAYVTPWSLMPLYGYWSRFV  197
Nlug_UV-der     115 NSFNLGFAAGHLGCQIFGTIGSFSGIGASATNAAIAYDRYNVIAKPFDG-KMSRGKAFLIILLIWAYVTPWSIMPLLEVWSRFV  197
Lstr_UV-der     115 NSFNLGFAAGHVGCQIFGTIGSFSGIGASATNAAIAYDRYNVIAKPFDG-KMSRGKAFLIILLIWAYVTPWSIIPLLEVWSRFV  197
Sfur_UV-der     115 NSFNLGFAAGHLGCQIFGTIGSFSGIGASATNAAIAYDRYNVIAKPFDG-KMSRGKAFLIILLIWAYVTPWSIMPLFEVWSRFV  197
```

```
                   11111111111111111111222222222222222222222222222222222  222222222          2  2222222
                   78888888888999999999900000000000111111111122222222222333  333333344          4  4444444
                   01234567890123456789012345678901234567890123456789012  345678901          2  3456789
Btau_rhodopsin 180 PEGMQCSCGIDYYTPHEETNNESFVIYMFVVHFIIPLIVIFFCYGQLVFTVKE--AAAQQQESA----------T--TQKAEKE  249
Craj_UV2-anc   192 PEGFLTQCTFDYLTNTFDT--HLFVGVLFTFSYVIPMSLIIYYYSQIVSHVIDHEKALREQAKKMNVDSLRSNQQQQNQSAEVR  273
Adif_UV2-anc   210 PEGYLTQCGIDYLTLTFDN--KSFVTTLFTCSYVIPMSLIIYYYIQIVGHVMDHEKALKEQAKKMNVDSLRSNQQQQNQSMEIR  291
Craj_UV1-der   197 PEGFLTACSFDYLTDSFDN--HLFVASIFTTSYVIPMLLIIYYYSQIVMHVIRHERTLKEQAKKMNVDSLRSNQTQMNQSTEIR  278
Adif_UV1-der   195 PEGFLTTCSFDYITDTFDT--RLFVVTIFVWSYVFPLLLIIYFYTQIVAHVFRHEKALKQQAKKMNVDSLRSNQAQADQSMEIR  276
Focc_UV        205 PEGFLTSCTFDYMSDTDHI--RVFVGVMFFVCYCIPMTMIIYFYSQIVSHVVAHEKALKAQAKKMNVESLRSNVDKNQDSTEIR  286
Dcit_UV        224 PEGYLTSCTFDYLTPTESI--RFYVLFMFIICYCIPMGMIINFYSQIVGHVFSHEKALREQAKKMNVESLRSGQKEGQSSAEVR  305
Pcel_UV        145 PEGYLTSCSFDYLTPTESV--RFYVLFMFIICYCFPMTLIINFYSQIVGHVFSHEKALREQAKKMNVESLRSGQKEGQSSAEVR  226
Apis_UV-anc    201 PEGYLTSCTFDYLSPTDET--RAFVGIMFVICYVIPVSLVIFFYSQIVSHVFNHEKALREQAKKMNVESLRSNQDANAQSAEVR  282
Rmai_UV-anc    201 PEGYLTSCTFDYLSPTDET--KAFVGIMFVICYVIPVSLVIFFYSQIVSHVFNHEKALREQAKKMNVESLRSNQDANAQSAEVR  282
Mper_UV-anc    201 PEGYLTSCTIDYLTPTDET--RAFIGIMFVICYVIPVSLVIFFYSQIVSHVFNHEKALREQAKKMNVESLRSNQDANAQSAEVR  282
Sfla_UV-anc    201 PEGYLTSCTIDYLTPTDET--RAFIGIMFTICYIIPVSLVIYFYSQIVSHVFNHEKALREQAKKMNVESLRSNQDANAQSAEVR  282
Cced_UV-anc    200 PEGYLTSCTFDYLSPTDET--KAFVGIMFVICYVIPMSLVVYFYSQIVTHVFNHEKALREQAKKMNVESLRSNQDANAQSAEVR  281
Apis_UV-der    196 PEGFLTSCSFDYLTPTEET--KAFVGTMFVICYVIPMSFIIYFYSQIVCHVFNHEKALREQAKKMNVESLRSNQDANAQSAEVR  277
Rmai_UV-der    196 PEGFLTSCSFDYLTPTEET--KAFVATMFVICYVIPMSFIIYFYSQIVCHVFNHEKALREQAKKMNVESLRSNQDANAQSAEVR  277
Mper_UV-der    196 PEGFLTSCSFDYLTPTEET--KAFVGTMFVICYVIPMSFIIYFYSQIVCHVFNHEKALREQAKKMNVESLRSNQDANAQSAEVR  277
Sfla_UV-der    194 PEGFLTSCSFDYLTPTDET--KAFVGILFVICYVIPMSFIIYFYSQIVSHVFNHEKALREQAKKMNVESLRSNQDANAQSAEVR  275
Cced_UV-der    194 PEGFLTSCSFDYLTQTDET--KFFVGVLFVICYVIPLSFIIYFYSQIVNHVFNHERALREQAKKMNVESLRSNQDANAQSAEVR  275
Ncin_UV        199 PEGYLTACSFDYLTNTDEN--RFFVLVLFCVCYVLPMSLIVFFYSQIVSHVVHHEKALRDQAKKMNVESLRSNQQQQSQSAEIR  280
Evit_UV        202 PEGYLTACSFDYLTNTDEN--RFFVLVLFFVCYVLPMSMIIFFYSQIVSHVVDHEKALREQAKKMNVESLRSNQSQQAQSAEIR  283
Monu_UV        202 PEGYLTACSFDYLTNTDEN--RFFVLVLFFVCYVLPMSMIIFFYSQIVSHVVDHEKALREQAKKMNVESLRSNQNQQAQSAEIR  283
Nlug_UV-anc    198 PEGYLTSCSFDYLTKSESI--QYWVGTMFVICYCIPMSLIIYYYSQIVSHVVNHEKALREQAKKMNVDSLRSNQAQQNQSAEVR  279
Lstr_UV-anc    198 PEGYLTSCSFDYLTKNESI--QYWVGTMFVICYCIPMSLIVYYYSQIVSHVVNHEKALREQAKKMNVDSLRSNQAQQNQSAEVR  279
Sfur_UV-anc    198 PEGYLTSCSFDYLTKNESI--QYWVGTMFVICYCIPMSMIIYYYSQIVSHVVNHEKALREQAKKMNVDSLRSNQAQQNQSAEVR  279
Nlug_UV-der    198 PEGFLTSCSFDYLTKTDSI--RYWTATMFVICYCIPMSMIIYYYSQIVSHVVNHEKALRKQAKKMNVDSLRSNQAAQSMSAEVR  279
Lstr_UV-der    198 PEGFLTSCSFDYLTNTDSI--RYFTATMFVICYCIPMSMIIYYYSQIVNHVVNHEKALRKQAKKMNVDSLRSNQAQQSMSAEVR  279
Sfur_UV-der    198 PEGFLTSCSFDYLTKTDSI--RYFTAVMFLICYCIPMSMIIYYYSQIVNHVVNHEKALRKQAKKMNVDSLRSNQAQQSMSAEVR  279
```

```
                     22222222222222222222222222222222222222222222222222333333333333333333333333333333333
                     55555555556666666666777777777788888888889999999999000000000011111111112222222222333
                     01234567890123456789012345678901234567890123456789012345678901234567890123456789012
Btau_rhodopsin  250  VTRMVIIMVIAFLICWLPYAGVAFYIFTHQGSDFGPIFMTIPAFFAKTSAVYNPVIYIMMNKQFRNCMVTTLCCGKNPLGDDE-  332
Craj_UV2-anc    274  IAKVAITICFLFVASWTPYAVLAMIGAFGDQSLITPGFAMIPACACKLVACLDPYVYALSHPRYRVELQKRLPWLAINEKDP--  355
Adif_UV2-anc    292  IAKVAITICFLFVAAWTPYGVMCLIGMYGDQNLITPGSAMVPALFCKTVACLDPYVYALSHPRFRLELQKRIPWLGINEKAP--  373
Craj_UV1-der    279  IAKVALTITFLYVAAWTPYAVVAMIGAFGDQSLLTPGITMIPSCFCKCVACLDPYVYALSHPRYRVELQKRLPWLAIVEPTQ--  360
Adif_UV1-der    277  IAKVAITITFLFVAAWTPYAVVSFIGAFGDRSLLTPGVTMIPACCCKFIACVDPYVYALSHPRYRVELQRRLPWLSIKEHVP--  358
Focc_UV         287  IAKMAISLSFLFVLSWTPYATVALIGAFGNKALLTPGVTMIPACCCKLVACFDPYVFALSHPRFRLELQKKMPFLDIKEDKPT-  369
Dcit_UV         306  IAKTAITLCALFVASWTPYAVVALTGAFGDQSLLTPGLTMIPACTCKAVACLDPYVYAISHPRYRLELSKRIPCLGIKEKEPE-  388
Pcel_UV         227  IAKTAITLCALFVFSWTPYAVVALTGAFGDRSLLTPGLTMIPACACKTVACLDPYVYAISHPRYRLELTKRFPCLGIKEKEPE-  309
Apis_UV-anc     283  IAKAAITICCLFIASWTPYAVVAMIGAFGDRSLLTPGITMIPAIFCKTVACFDPYVYAISHPRYRLELSKRVPCLGISEKPPPT  366
Rmai_UV-anc     283  IAKAAITICCLFIASWTPYAIVAMIGAFGDRSLLTPGITMIPAIFCKTVACFDPYVYAISHPRYRLELSKRVPCLGINEKPPPT  366
Mper_UV-anc     283  IAKAAITICCLFIASWTPYAVVAMIGAFGDRSLLTPGVTMIPAIFCKTVACFDPYVYAISHPRYRLELSKRVPCLGINEKPPT-  365
Sfla_UV-anc     283  IAKAAITICFLFIAAWTPYAVVAMIGAFGDQSLLTPGITMIPAIFCKSVACFDPYVYAISHPRYRLELSKRIPCLGIEEKPPAT  366
Cced_UV-anc     282  IAKAAITICFLFIAAWTPYAVVAMIGAFGDKSLLTPGVTMIPAIFCKTVACFDPYVYAISHPRYRLELSKRIPCLGINEKPPAT  365
Apis_UV-der     278  IAKAAITICFLFVAAWTPYAVVAMIGAFGDQSLLTPIASMLPAVFAKTVACFDPYVYAISHPKYRLELSKRVPCLGITEKPLA-  360
Rmai_UV-der     278  IAKAAITICFLFVAAWTPYAIVAMIGAFGDQSLLTPIASMLPAVFAKTVACFDPYVYAISHPKYRLELSKRVPCLGINEKPPA-  360
Mper_UV-der     278  IAKAAITICFLFVAAWTPYAVVAMIGAFGDQSLLTPIASMLPAVFAKTVACFDPYVYAISHPKYRLELSKRVPCLGITEKPPA-  360
Sfla_UV-der     276  IAKAAITICFLFVAAWTPYAVVAMVGAFGDQSLLTPVVTMLPAVFAKTVACLDPYVYAISHPKYRLELSKRVPCLGINEKSSV-  358
Cced_UV-der     276  IAKAAITICFLFIAAWTPYAAVALIGAFGDQSLLTPIVTMLPAVFAKTVACFDPYVYAISHPRYRLELSKRLPCLGINEKPPV-  358
Ncin_UV         281  IAKAAITICFLFVASWTPYAVLALIGAFGDQSLLTPGVTMIPALTCKAVACIDPYVYAISHPRYRVELAKRLPFLAINEQAP--  362
Evit_UV         284  IAKAAITICALFVASWTPYAVLALIGAFGNQAMLTPGVTMIPALTCKAVARIDPYVYAISHPRYRVELAKRLPWLGVNEQEPK-  366
Monu_UV         284  IAKAAITICALFVASWTPYAVLALIGAFGNQAMLTPGVTMIPALTCKAVACIDPYVYAISHPRYRVELAKRLPWLGVNEQEPK-  366
Nlug_UV-anc     280  IAKVAITICFLFVASWTPYAVVAMIGAFGNQALLTPGLTMIPACTCKAVACIDPYVYAISHPRYRVELAKRLPWLAIKEAAPV-  362
Lstr_UV-anc     280  IAKVAITICFLFVASWTPYAVVAMIGAFGNQALXTPGLTMIPACTCKAVACIDPYVYAISHPRYRVELAKRLPWLAIKEAPPP-  362
Sfur_UV-anc     280  IAKVAITICFLFVASWTPYAVVAMIGAFGNQALLTPGLTMIPACTCKAVACIDPYVYAISHPRYRVELAKRLPWLAIKEAPPP-  362
Nlug_UV-der     280  IAKVAITICALFVAAWTPYAIVALIGAFGNQSLLTPGVTMIPALACKSVACIDPYVYAISHPRYRMELAKRLPWLKIKETPTA-  362
Lstr_UV-der     280  IAKVAITICFLFVAAWTPYAIVALIGAFGNQSLLTPVVTMIPALACKSVACIDPYVYAISHPRYRVELAKRLPWLRIKETSAA-  362
Sfur_UV-der     280  IAKVAITICFLFVAAWTPYAIVALIGAFGNQSLLTPVVTMIPALACKSVACIDPYVYAISHPRYRVELAKRIPWLRIKETPTA-  362
```

```
                      33333333333 33 333
                      33333334444 44 444
                      34567890123 45 678
Btau_rhodopsin 333 -ASTTVSKTETS-QV-APA-------  348
Craj_UV2-anc   356 -SDTQSTATESS-TN-K---P-----  370
Adif_UV2-anc   374 -SDAQSVTTETA-SK-----G-----  387
Craj_UV1-der   361 -TDTASAVTEAS-NP-ATR-T-----  377
Adif_UV1-der   359 -SDSASVATESA-AP-NTGKI-----  376
Focc_UV        370 ASETASTTTEAT-SA-NPD-------  386
Dcit_UV        389 -KETASAQTEAT-T---P-------H  402
Pcel_UV        310 -KETASANTEAT-T---P-------H  323
Apis_UV-anc    367 ASETQSTTTA---------------A  377
Rmai_UV-anc    367 ASETQSTTTTT--------------A  378
Mper_UV-anc    366 PSETQSTTTTT--------------A  377
Sfla_UV-anc    367 ASETQSTTTTT--------------A  378
Cced_UV-anc    366 VSETQSATTT---------------A  376
Apis_UV-der    361 TSDTQSITTA---------------A  371
Rmai_UV-der    361 TSDTQSITTA---------------A  371
Mper_UV-der    361 TSDTQSITTA---------------A  371
Sfla_UV-der    359 SPDTQSTTTT---------------A  369
Cced_UV-der    359 LAETQSTTTS---------------A  369
Ncin_UV        363 SE-TVSTTTETTAA-TTPA------E  380
Evit_UV        367 HDNVSTTTTEAT-A-TTPA------E  384
Monu_UV        367 HDNVSTTTTEAT-A-TTPA------E  384
Nlug_UV-anc    363 -SETQSAVTETT-AS-TPAES-----  380
Lstr_UV-anc    363 -SETQSAVTETT-AT-TPAE------  379
Sfur_UV-anc    363 -SETQSAVTETT-AT-TPAE------  379
Nlug_UV-der    363 -SETQSTVTEST-VTATPAE------  380
Lstr_UV-der    363 -SETQSTVTEST-VTAPPAE------  380
Sfur_UV-der    363 -SETQSTVTEST-VSATPAVTATECN  386
```
